# High throughput measurement of metabolism in planarians reveals activation of glycolysis during regeneration

**DOI:** 10.1002/reg2.95

**Published:** 2018-01-11

**Authors:** Edie A. Osuma, Daniel W. Riggs, Andrew A. Gibb, Bradford G. Hill

**Affiliations:** ^1^ Institute of Molecular Cardiology, Diabetes and Obesity Center, Department of Medicine University of Louisville Louisville KY USA; ^2^ Diabetes and Obesity Center, Department of Medicine University of Louisville Louisville KY USA; ^3^ Wesleyan College Macon GA USA; ^4^ Department of Physiology University of Louisville Louisville KY USA

**Keywords:** glycolysis, metabolism, mitochondria, planaria, respiration

## Abstract

Planarians are outstanding models for studying mechanisms of regeneration; however, there are few methods to measure changes in their metabolism. Examining metabolism in planarians is important because the regenerative process is dependent on numerous integrated metabolic pathways, which provide the energy required for tissue repair as well as the ability to synthesize the cellular building blocks needed to form new tissue. Therefore, we standardized an extracellular flux analysis method to measure mitochondrial and glycolytic activity in live planarians during normal growth as well as during regeneration. Small, uninjured planarians showed higher rates of oxygen consumption compared with large planarians, with no difference in glycolytic activity; however, glycolysis increased during planarian regeneration. Exposure of planarians to koningic acid, a specific inhibitor of glyceraldehyde‐3‐phosphate dehydrogenase, completely abolished extracellular acidification with little effect on oxygen consumption, which suggests that the majority of glucose catabolized in planarians is fated for aerobic glycolysis. These studies describe a useful method for measuring respiration and glycolysis in planarians and provide data implicating changes in glucose metabolism in the regenerative response.

## 1 INTRODUCTION

Planarians are useful models for understanding the mechanisms of tissue regeneration. These bilaterally symmetrical metazoans are found in freshwater streams and ponds and have been used in regeneration studies since the early 19th century (Elliott & Sanchez Alvarado, 2013). Perhaps most notable are studies in the late 1890s by Thomas H. Morgan showing that small fragments comprising only 1/279 of the intact planarian are capable of regenerating a complete worm (Morgan, 1898). The mechanisms responsible for such regenerative capacity are of strong interest because they could shed light on understanding why some organs, such as the adult mammalian heart and brain, lack the ability to regenerate after injury. Knowledge of these mechanisms could contribute to therapeutic strategies to promote tissue repair in regeneration‐obstinate organs.

In planarians, regeneration requires proliferation and differentiation of stem cells, known as neoblasts, as well as growth and reorganization of existing tissue, termed morphyllaxis (Reddien & Sanchez Alvarado, 2004). These processes are probably associated with changes in intermediary metabolic pathways that meet the increased bioenergetic demand for growth and repair. Changes in metabolism would also be important for increasing the biosynthesis of nucleotides, phospholipids, and amino acids, thereby constituting a material cause for regeneration (Noor, Eden, Milo, & Alon, 2010). Although early studies by Morgan showed that starvation and feeding alter regeneration (Morgan, 1900), surprisingly little regarding the metabolic regulation of planarian regeneration has been documented, which is due in part to limitations in methodology. Therefore, we standardized a high throughput method to measure in planarians oxygen consumption and extracellular acidification, which are indicative of mitochondrial activity and glycolysis, respectively. Using this method, we further characterized how metabolism changes after injury and during early regeneration. This method will be useful for understanding how specific genes regulate metabolism in planarians and how metabolism coordinates planarian tissue repair and regrowth.

## 2 RESULTS

### 2.1 Standardization of planarian metabolic assay conditions

**Figure 1:**
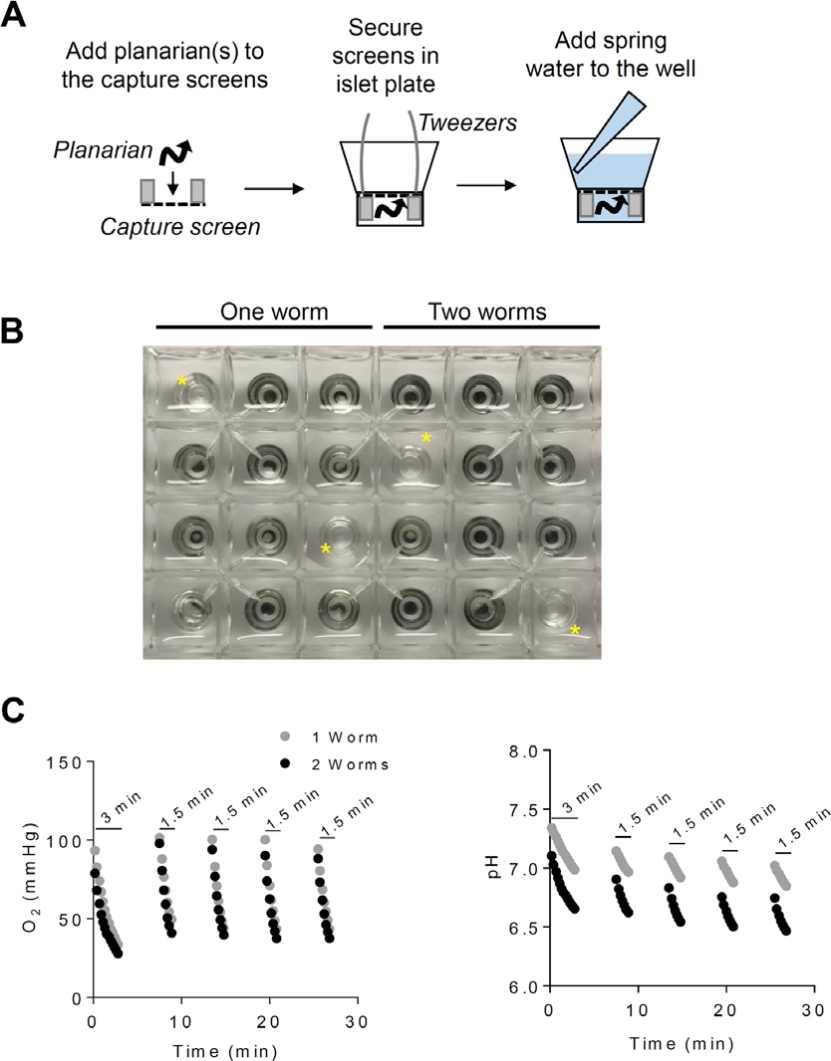
Development of the protocol to measure planarian metabolism by XF analysis. (A) Schematic showing the workflow for securing planarians into an XF24 islet plate prior to metabolic measurements in the XF24 instrument. (B) Image of a plate “seeded” with one or two planarians per well. The yellow asterisks (*) indicate blank wells that served as temperature control wells. (C) O_2_ and pH traces acquired during 3 min and 1.5 min measure times.

Although extracellular flux (XF) analysis has been used to measure metabolism in *Caenorhabditis elegans* (Luz, Smith, Rooney, & Meyer, 2015; Luz et al., 2015) and tissue explants (Cummins et al., 2014; Sansbury et al., 2012), the technology has not been standardized for examining planarian metabolism. Therefore, in an initial test to determine whether the technology can work for measuring planarian metabolism, we placed one or two planarians (∼2 cm in length) into islet capture screens, which were then secured into the XF24 pancreatic islet microplate. The islet screens prevent planarians from escaping from the bottom of the well. After securing the capture screen within the well, 750 μL of fresh spring water was added to the well; this was repeated until all wells were “seeded” with planarians (Fig. 1A, B).

After calibration of the XF24 instrument, we measured planarian oxygen consumption and changes in pH using a standard protocol, which involved 2 min mix, 2 min wait, and 3 min measure cycles. As shown in the representative traces in Figure 1(C), changes in the oxygen concentration during the first measurement were not linear, and the oxygen levels dropped below 25 mmHg. To prevent hypoxic conditions, we adjusted the measure time to 1.5 min, which provided more linear oxygen traces and prevented near hypoxic conditions from occurring during subsequent measurements. It should be noted that, for calculating oxygen consumption rates (OCRs), we used the Akos correction algorithm (Gerencser et al., 2009). This algorithm eliminates artifactual decay of the raw OCR trace such that linear rates are not essential for accurately assessing oxygen consumption during the measurement. Of note, most software for current XF24e instruments requires a minimum of a 2 min read time, which we found in later experiments to be sufficient for measuring OCRs, while at the same time preventing oxygen concentrations from dropping too low during the measurements (see Fig. S1).

Collectively, these initial experiments illuminate testing parameters that can be used in the XF platform to examine OCR in planarians. Our findings indicate that two worms per well cause excessive crowding, which could affect planarian activity and metabolism, and they demonstrate that one worm per well is sufficient for obtaining detectable rates of oxygen consumption.

### 2.2 Small planarians demonstrate higher normalized oxygen consumption

**Figure 2:**
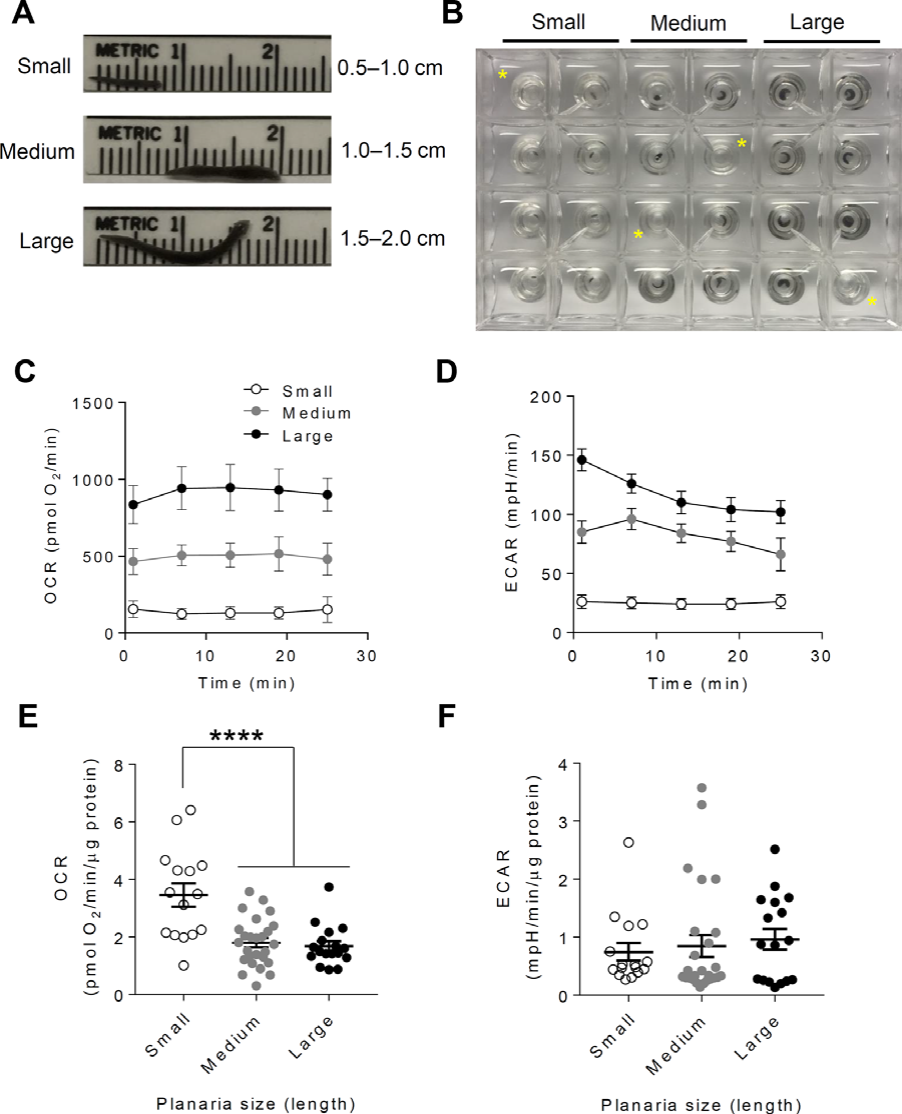
Small planarians consume more oxygen than large planarians. Extracellular flux analysis of planarians of different sizes. (A) Planarians grouped according to length. (B) Representative image of XF24 islet plate seeded with planarians of different sizes. The yellow asterisks (*) indicate blank wells that served as temperature control wells. (C), (D) Representative traces showing the unnormalized oxygen consumption rate (OCR) and extracellular acidification rate (ECAR), respectively; (E), (F) OCR and ECAR values normalized to protein. Due to failure of normality, data for (F) were log‐transformed prior to statistical analysis. *n* = 15–26 worms per group. ****P < 0.0001 (one‐way ANOVA followed by Dunnett's correction).

We next determined the effect of worm size on metabolic activity. For this, we segregated the planarians in the XF24 plates based on their length, with small planarians classified as 0.5–1.0 cm; medium planarians classified as 1.0–1.5 cm; and large planarians being 1.5–2.0 cm (Fig. 2A, B). Measurement of the OCR showed the expected size‐dependent increases in OCR, with small, medium, and large planarians showing a mean OCR of ∼130, 510, and 950 pmol O_2_/min (Fig. 2C). The raw extracellular acidification rate (ECAR) values showed a similar trend (Fig. 2D). To normalize the values to biomass, we measured protein content in each worm. As shown in Figure S2, protein content was highly correlated with gravimetric measurements of the planarians. Once normalized to total protein, small planarians showed 2‐fold higher OCR compared with medium and large worms (Fig. 2E), which is consistent with previous findings (Allen, 1919; Hyman, 1919c) and helps validate this as a high throughput method to measure OCR in planarians. Size‐dependent effects on ECAR were not evident (Fig. 2F).

### 2.3 Effect of injury and regeneration on OCR and ECAR in planarians

**Figure 3:**
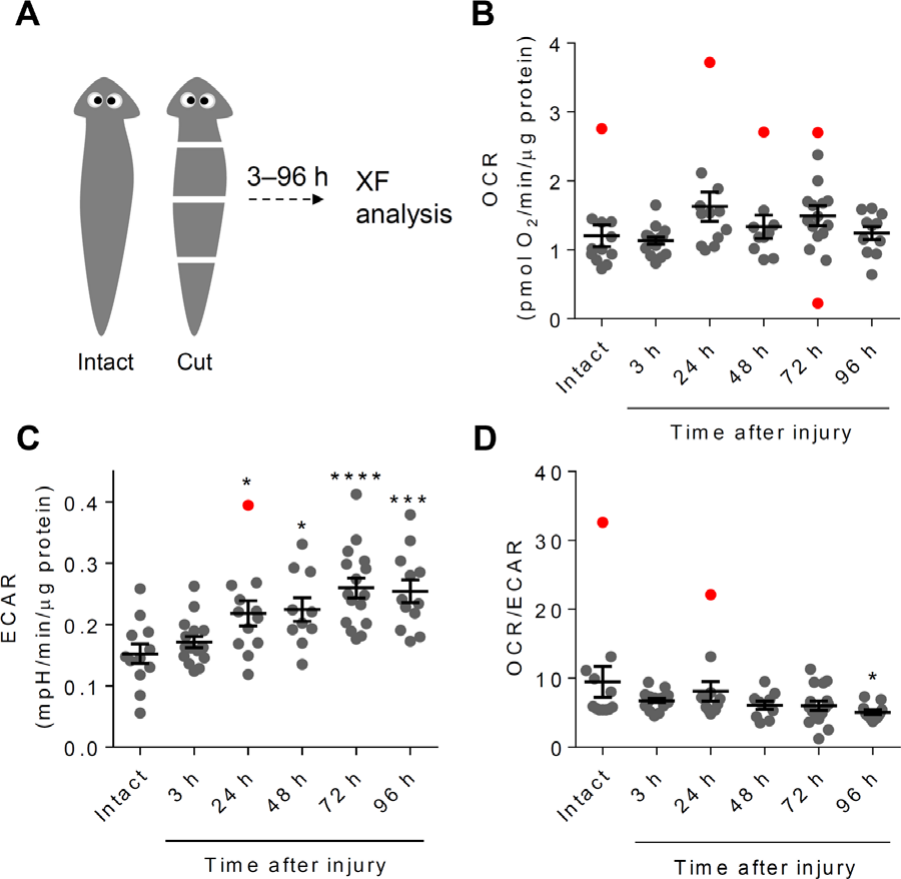
Extracellular acidification is increased during regeneration. Extracellular flux analysis in intact, acutely injured, and regenerating planarians. (A) Worms were segmented into fourths 3–96 h before XF analysis. (B) Oxygen consumption rate (OCR) in intact, acutely injured, and regenerating planarians. The rates were normalized to total protein. (C) Normalized extracellular acidification rate (ECAR) values in intact, acutely injured, and regenerating worms. (D) The ratio of OCR to ECAR, which is an indicator of reliance on oxidative metabolism versus glycolysis. *n* = 10–16 worms per group. *P < 0.05, **P < 0.001, ***P < 0.001, ****P < 0.0001 (one‐way ANOVA followed by Dunnett's correction) versus intact worms. Points shown in red were ±2SD from the mean. Data in which outliers were removed are shown in Figure S3.

To determine whether metabolism changes during regeneration, we segmented the planarians into four sections and measured OCR and ECAR 3–96 h after injury. For these experiments, planarians designated for all groups were fed on Friday. Lateral cuts were made in a reverse time course manner on Monday, Tuesday, Wednesday, and Thursday for the 96 h, 72 h, 48 h, and 24 h groups, respectively. An additional group was segmented 3 h prior to the XF assay, which was performed on Friday (Fig. 3A). Intact control specimens and the segmented pieces were then placed into their respective wells, similar to that shown in Figure 1(A). As shown in Figure 3(B), we found that OCR was not significantly different after injury; however, we noticed that several points were more than two standard deviations from the mean, which could qualify them as statistical outliers. As shown in Figure S3(A), once these outliers were removed, the OCR in the 24 h and 72 h injured groups appeared significantly higher than in the intact group.

The ECAR was significantly elevated in a time‐dependent manner in all groups that were segmented 24–96 h prior to the assay (Fig. 3C). After removal of the one statistical outlier from this dataset, the 24 h regenerating group lost statistical significance (Fig. S3B). With time after injury, there was a decreasing trend in the OCR:ECAR values, with a significant difference occurring 96 h after injury; however, no significant differences in OCR:ECAR were found after removal of statistical outliers (Figs 3D and S3C).

### 2.4 Effect of metabolic inhibitors on extracellular flux measurements

Metabolic inhibitors are commonly used in XF assays to interrogate metabolism in isolated cells (Dranka et al., 2011; Hill, Dranka, Zou, Chatham, & Darley‐Usmar, 2009; Hill et al., 2012). To determine whether metabolic inhibitors could be used for this purpose in planarians, we exposed the worms to inhibitors of mitochondrial electron transport or glycolysis and measured changes in OCR and ECAR. The inhibitors were loaded into the ports at a 10× concentration, which upon injection achieved the desired (1×) concentration. Rotenone, a complex I inhibitor, used at final concentrations up to 50 μmol/L (500 μmol/L port concentration) led to only modest inhibition of respiration within the time frame of the measurements, and it had no effect on ECAR (Fig. S4A). We encountered similar difficulties with antimycin A, a commonly used complex III inhibitor. Concentrations up to 50 μmol/L failed to inhibit mitochondrial respiration effectively (Fig. S4B). The testing of higher concentrations is constrained by the limited water solubility of these compounds; however, exposure of planarians to the complex IV inhibitor potassium azide decreased OCR in a concentration‐dependent manner, with ∼60% inhibition of OCR achieved with 100 mmol/L azide within 50 min of injection. Potassium azide also showed prominent effects on ECAR. The 1 mmol/L concentration acutely increased ECAR, whereas higher concentrations (10 and 100 mmol/L) rapidly inhibited ECAR, followed by a period of slow recovery of apparent glycolysis (Fig. S4C).

Although glycolysis is the prominent source of protons extruded from cells and tissues, protons are also generated during respiration via pyruvate‐dehydrogenase‐mediated production of CO_2_ (i.e., CO_2_ → HCO_3_^−^ + H^+^) (Mookerjee, Goncalves, Gerencser, Nicholls, & Brand, 2015; Mookerjee, Gerencser, Nicholls, & Brand, 2017). Our previous studies show that koningic acid, a potent and irreversible inhibitor of glyceraldehyde‐3‐phosphate dehydrogenase (Endo, Hasumi, Sakai, & Kanbe, 1985; Sakai, Hasumi, & Endo, 1988, 1991), can completely inhibit extracellular acidification in isolated mammalian cells (Salabei et al., 2015; Sansbury et al., 2011). Similarly, exposure of planarians to 1 mmol/L koningic acid quickly and completely abolished extracellular acidification without much change in OCR (Fig. S4D). These data suggest that koningic acid is suitable for inhibiting glycolysis in planarians. In addition, the finding that koningic acid only minimally affects OCR despite complete inhibition of ECAR suggests that aerobic glycolysis is the major route of glucose catabolism in planarians.

### 2.5 Increases in ECAR during planarian regeneration are due to glycolysis

**Figure 4:**
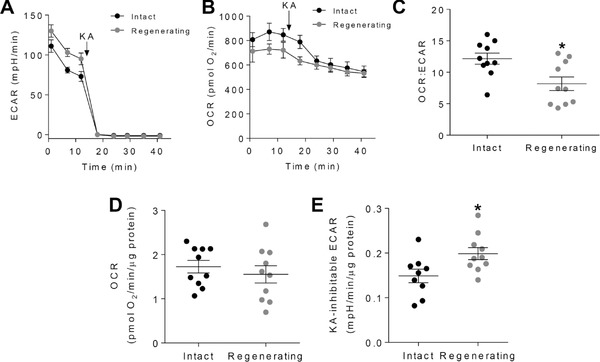
Glycolysis is increased in regenerating planarians. Extracellular flux analysis of intact planarians and planarians that were segmented into fourths 72 h before assay. (A) Extracellular acidification rate (ECAR) in intact and regenerating planarians. As a control for later time points, one sample in the intact group did not receive koningic acid (KA). Shown are only those samples that received KA. (B) Oxygen consumption rate (OCR) in intact and regenerating planarians. Shown are only those samples that received KA. (A), (B) KA (0.7 mmol/L final concentration) was injected after the third baseline measurement. (C) Ratio of OCR to ECAR derived from all samples, prior to KA injection. (D) Normalized OCR in intact and regenerating planarians. (E) Normalized ECAR in intact and regenerating planarians. *n* = 9–10 per group. *P < 0.05 (unpaired Student's *t* test).

To determine whether or not the increases in ECAR shown to occur during regeneration are due to augmentation of glycolysis, we measured metabolism after planarian segmentation, in the absence or presence of koningic acid. We chose the 72 h time point after injury because it had previously shown the largest increase in glycolysis (see Fig. 3C). Raw ECAR traces showed higher apparent ECAR, which was fully inhibited upon addition of koningic acid (Fig. 4A). In contrast, raw OCR traces showed that intact planarians showed higher apparent OCR (Fig. 4B). The addition of koningic acid had a relatively small effect on oxygen consumption in both groups, with OCR in the regenerating group being least affected by the compound. Prior to exposure of the worms to koningic acid, the ratio of OCR to ECAR was significantly lower in regenerating planarians (Fig. 4C), indicating higher reliance on glycolysis during regeneration. Although OCR normalized to protein was not different between intact and regenerating planarians (Fig. 4D), regenerating planarians showed significantly higher koningic‐acid‐inhibitable ECAR (Fig. 4E). These data demonstrate that the higher rates of extracellular acidification observed in regenerating planarians are due to activation of glycolysis.

## 3 DISCUSSION

In this study, we standardized a high throughput method to measure both oxygen consumption and glycolysis in planarians. This method facilitates use of the XF24 analyzer, which is common in most research environments. The salient conclusions of our study are that small planarians use more oxygen than larger planarians and that the early stages of regeneration in planarians are associated with increases in glycolytic rate. Power calculations based on the data shown here provide a guide for predicting the number of worms required to detect statistically significant differences in metabolism. We provide initial recommendations based on our data in Tables S1 and S2. From these calculations, we predict that 10–11 planarians per group should provide the ability to distinguish a 50% difference in OCR or ECAR at 80% power and with an *α* of 0.05.

Previous methods for measuring respiration and glycolysis were limited by their low throughout nature, their requirement for a large amount of biomass, and/or their indirect methods of measurement. For example, past methods to measure oxygen consumption in planarians include the Winkler method for dissolved oxygen (e.g., Allen, 1919; Hyman, 1919a), the susceptibility method (Child, 1914, 1919), and manometric methods (Fraps, 1930), such as Cartesian diver respirometry (Pedersen, 1956). Early studies using these techniques focused primarily on changes in oxygen consumption due to feeding, starvation, worm size, injury, and regeneration (Allen, 1919; Hyman, 1919a, 1919b, 1919c, 1920, 1923; Hyman, Willier, & Rifenburgh, 1924). Considerably less research on glycolysis in planarians has been attempted, which is probably due to technical limitations. A method described by Fraps suggested the determination of glycolysis in planarians based on the liberation of CO_2_ from NaHCO_3_ (in Ringer solution) in the presence of grape sugars (Fraps, 1930); however, NaHCO_3_ was found to depress oxygen consumption, which would probably obfuscate veritable measurements of planarian metabolism. Other reports demonstrate methods to assess carbohydrate metabolism by measuring glucose or glycogen content in the worms or by measuring the activity of lactate dehydrogenase in lysates (Brand, 1936; Torres‐Da Matta, Kanaan, & Da Matta, 1989), all of which provide a limited view of glycolytic flux.

Findings congruent with those from previous studies help to validate the current method. For example, our finding that oxygen consumption is higher in small planarians compared with medium and large planarians is consistent with early studies by Allen (1919) and Hyman (1919c). Additionally, the finding that OCR was not remarkably or consistently different in intact versus regenerating planarians is consistent with several previous studies (Hyman, 1923; Lovtrup, 1953; Pedersen, 1956; Vladimirova, 1981). It should be stated that we did not assess OCR late in regeneration (i.e., more than 4 days after injury), a time in which increased respiration has been documented (Vladimirova, 1981). Thus, our findings do not rule out the possibility that respiration may change more profoundly at later stages of planarian regeneration.

Because oxygen consumption was not remarkably different between the groups, we did not interrogate mitochondrial respiration further using inhibitors of the electron transport chain; however, we did assess the (un)suitability of several known inhibitors of respiration for the current method. The typical inhibitors used for XF assays include inhibitors of complexes I, III, IV, and V (Hill et al., 2012). We found both rotenone and antimycin A to be poor inhibitors of respiration in planarians, at the concentrations that are soluble in water. In additional studies, we found that providing 5% dimethyl sulfoxide (DMSO) in the well improved the solubility of both rotenone and antimycin A, allowing 100 μmol/L of each compound to be used; however, this led to only a 50% inhibition of OCR (data not shown). Higher concentrations of DMSO were toxic to the planarians and caused death. The best inhibitor of mitochondrial metabolism that we tested was potassium azide, which led to ∼60% inhibition of respiration within 1 h of administration. Nevertheless, azide markedly inhibited OCR only at high concentrations (100 mmol/L). Interestingly, treatment of planarians with 1 mmol/L azide increased ECAR, while higher concentrations led to rapid decreases in ECAR. The changes in ECAR with 1 mmol/L azide could be due to allosteric feedback (e.g., by AMP, ADP, and glycolytic intermediates at key nodes in glycolysis such as at the phosphofructokinase and pyruvate kinase steps) caused by modest inhibition of mitochondrial respiration. Higher concentrations of potassium azide had an opposite effect on glycolysis; however, the concentrations of potassium azide used are quite high, and the effects at the higher concentrations could be due in part to large changes in extracellular potassium concentration or off‐target effects. It is possible that the mucus or slime coat present on planarians limits exposure to metabolic agents or that the inhibitors tested do not inhibit electron transport as effectively in planarians as they do in mammalian cells. Finding inhibitors that quickly inhibit planarian respiration at lower concentrations, or development of mucolytics that improve entry of respiratory chain inhibitors into planarians without otherwise affecting metabolism, would further improve upon the ability to interrogate mitochondrial bioenergetics using this assay.

The major new finding of our study is that glycolytic flux is higher during regeneration. Our results suggest that glycolysis is maximally activated 72 h after injury, which coincides with the nexus between the “regeneration response” and the “early differentiation” phases of planarian regeneration (Wurtzel et al., 2015). This finding is consistent with previous observations of elevated glycolytic proteins during head regeneration (Chen & Xu, 2015). It is unlikely that extracellular acidification from planarians is due to non‐glycolytic sources of protons (Mookerjee et al., 2015, 2017) because koningic acid was able to prevent acidification fully and quickly. Moreover, the fact that koningic acid can completely inhibit extracellular acidification without much change in oxygen consumption suggests that the majority of glucose catabolized in planarians occurs via aerobic glycolysis, with relatively little glucose‐derived carbon used for glucose oxidation. It is likely that increases in glycolytic rate may be required not only for augmenting ATP production during regeneration, but also for regulating the allocation of glucose‐derived carbon for biosynthesis of the nucleotides, phospholipids, and amino acids needed for new tissue growth (Gibb et al., 2017).

There are several limitations to our study. Previous studies show that anterior and posterior fragments differ in their metabolism after injury (Child, 1914; Vladimirova, 1981); hence, our approach to measure metabolism in all tissue fragments derived from each planarian simultaneously may have obscured changes that occur in particular tissue regions. Also, because inhibitors of electron transport did not have remarkable effects on OCR, we did not thoroughly test whether the complex IV inhibitor oligomycin, which could be used to examine proton leak, or whether uncouplers of respiration are suitable for planarian metabolism studies; these could be tested and perhaps incorporated into assays in the future. Last, it should be noted that temperature control in the XF24 instrument is not as stable or accurate when adjusted to 25°C, as opposed to the more common setting of 37°C. In our studies, the temperature fluctuated between 26.0 and 27.3°C, which is slightly higher than the more common temperature for maintaining planarians, i.e., 22–25°C. Regardless, the slightly higher temperature during the assay did not appear to affect the results, as we were able to reproduce previously documented findings regarding the effect of planarian size and injury on OCR (Allen, 1919; Hyman, 1919c, 1923; Lovtrup, 1953; Pedersen, 1956; Vladimirova, 1981). Should lower temperatures be required, the XF24 instrument can be placed in a cooler environment (e.g., <16°C), which would enable the instrument to maintain a temperature of 22–25°C more accurately and stably.

In summary, we provide a method to measure planarian metabolism using the XF24 analyzer. This method should extend to most species of planarians. We find that although respiration did not change markedly during the early phases of regeneration, glycolysis increased in a time‐dependent manner after injury. Given the role of metabolism in maintaining stemness and regulating the differentiation of numerous types of stem cells (Agathocleous & Harris, 2013; Folmes & Terzic, 2016; Gaspar et al., 2014; Salabei et al., 2015), these findings suggest that metabolic changes in planarians probably play a role in governing regeneration. Refinement of our understanding of how glucose metabolism modulates regeneration could provide exciting opportunities to improve tissue repair capacity in mammalian organs.

## 4 MATERIALS AND METHODS

### 4.1 Materials and reagents

Brown planaria (*Dugesia dorotocephala*) were purchased from Carolina Biological Supply (Burlington, NC). All chemical reagents were from Sigma‐Aldrich (St Louis, MO), unless otherwise noted. Koningic acid (also known as heptelidic acid) was from Cayman Chemical Company (Ann Arbor, MI). Pancreatic islet plates and Seahorse XF flux packs were from Agilent Technologies (Santa Clara, CA).

### 4.2 Planarian culture, handling, and preparation for assay

The planarians were housed in 10‐cm Petri dishes filled with 20 mL of spring water and maintained at room temperature. The water was changed daily, excluding weekends. The worms were fed once a week, 3–7 days prior to assay, during which time they were provided hardboiled egg yolk for 1 h. The worms were then placed in clean Petri dishes and provided with fresh water.

### 4.3 Planarian injury and regeneration studies

Three days after feeding, planarians (1.5–2 cm in length) were cut laterally into four sections, and the fragments were placed into individual wells of six‐well Petri dishes containing fresh spring water. Metabolic assays were conducted 3–96 h after injury. Intact worms were used as controls.

### 4.4 Extracellular flux assay for planarians

Cellular energetics in intact planarians were measured using a Seahorse Bioscience XF24 extracellular flux analyzer and a modified protocol (modified from Cummins et al., 2014; Sansbury et al., 2012). All experiments were performed with the XF24 analyzer set to a temperature of 25°C. The planarians were first sequestered underneath capture screens in an XF24 islet plate. The screens were then secured in place in the bottom of each well, and 750 μL of fresh spring water was added. The worms were left undisturbed for 30 min, during which time the XF calibration program was completed; spring water was used as the calibration solution. After calibration, the plate containing the planarians was placed in the instrument for measurements of oxygen consumption and extracellular acidification. Basal OCRs and ECARs were measured during a programmed protocol: three cycles of 2 (or 3) min mix, 2 min wait, and 1.5–3 min measure times. Respiratory chain inhibitors were injected from the ports of the flux cartridge as described more fully in the Results. To normalize OCR and ECAR values, we measured the total protein content of each planarian using the Lowry DC Protein Assay kit (Bio‐Rad Laboratories, Hercules, CA). Briefly, after completion of the XF assay, the worms were removed from the wells and sonicated in 200 μL of water containing 2% sodium dodecyl sulfate (w/v). Following centrifugation for 10 min at 10,000*g*, the Lowry DC Protein Assay kit was used to measure protein content. The OCR and ECAR measurements were then normalized to total protein.

### 4.5 Statistical analysis

All values are mean ± SEM. Comparisons were made to the relevant control group and performed using either the unpaired Student's *t* test or one‐way ANOVA with Dunnett's correction for multiple comparisons. For statistical analysis of data that failed normality, the data were first log‐transformed, followed by the appropriate statistical test. The null hypothesis was rejected for *P* < 0.05.

## Supporting information

Figure S1

Figure S2

Figure S3

Figure S4

Tables S1 and S2
