# Supplementary figures and images for: High throughput measurement of metabolism in planarians reveals activation of glycolysis during regeneration
Source: Regeneration (Oxf). 2018 Jan 11;5(1):78–86. doi: 10.1002/reg2.95 (PMC5911454; doi:10.1002/reg2.95)

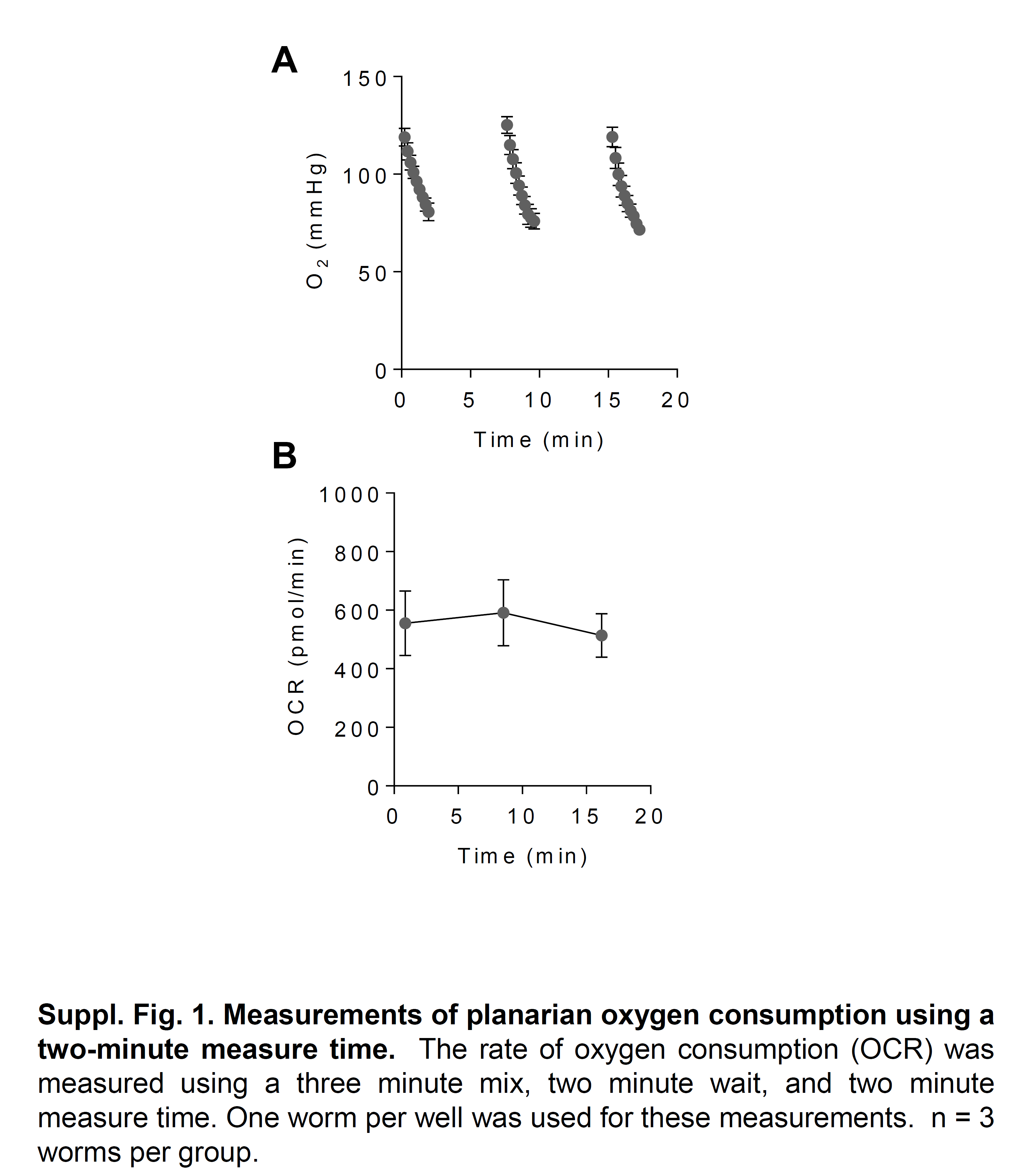

Supplement: Supplementary file 1 — Figure S1 [file REG2-5-78-s001.tif]

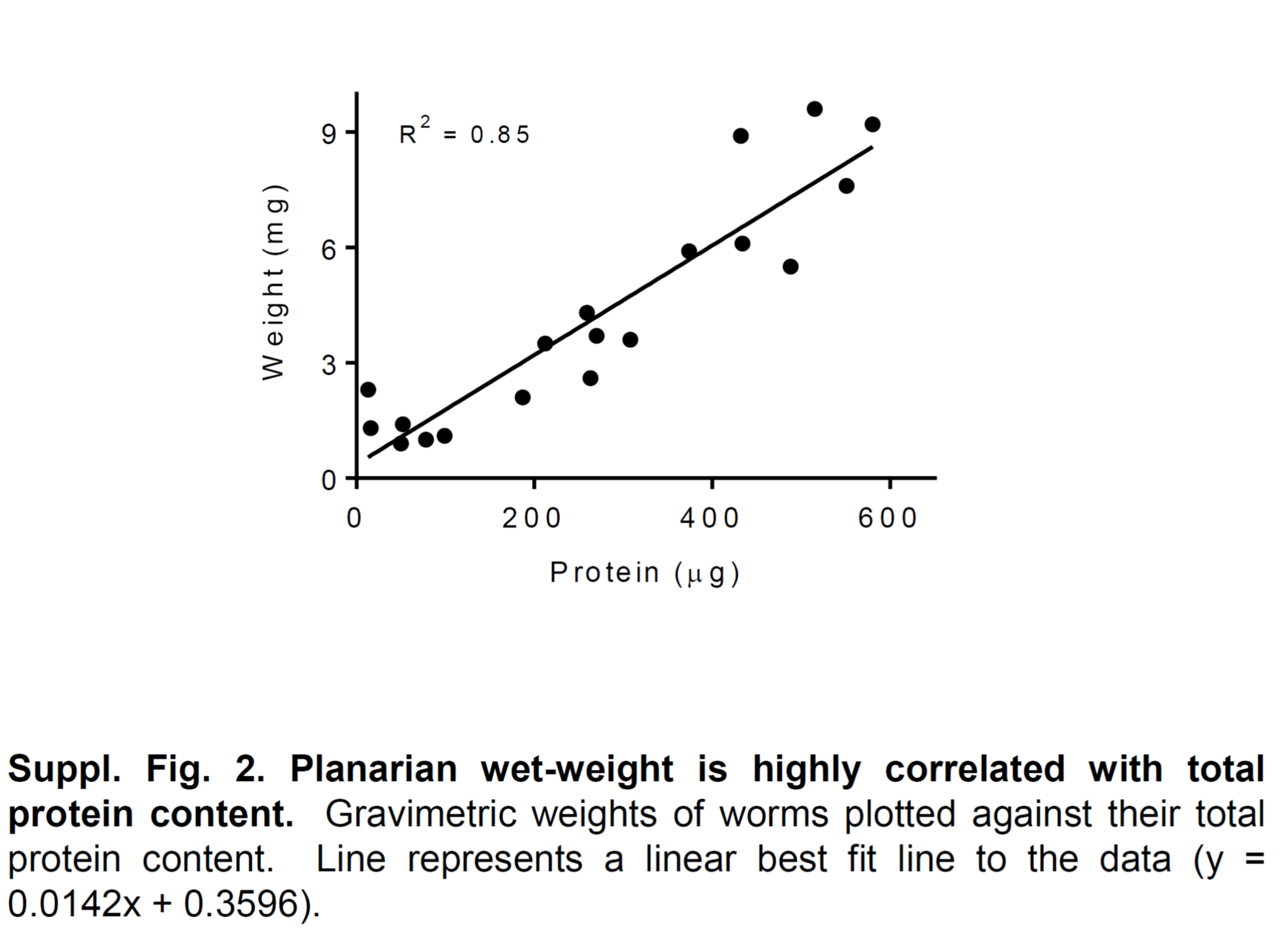

Supplement: Supplementary file 2 — Figure S2 [file REG2-5-78-s002.tif]

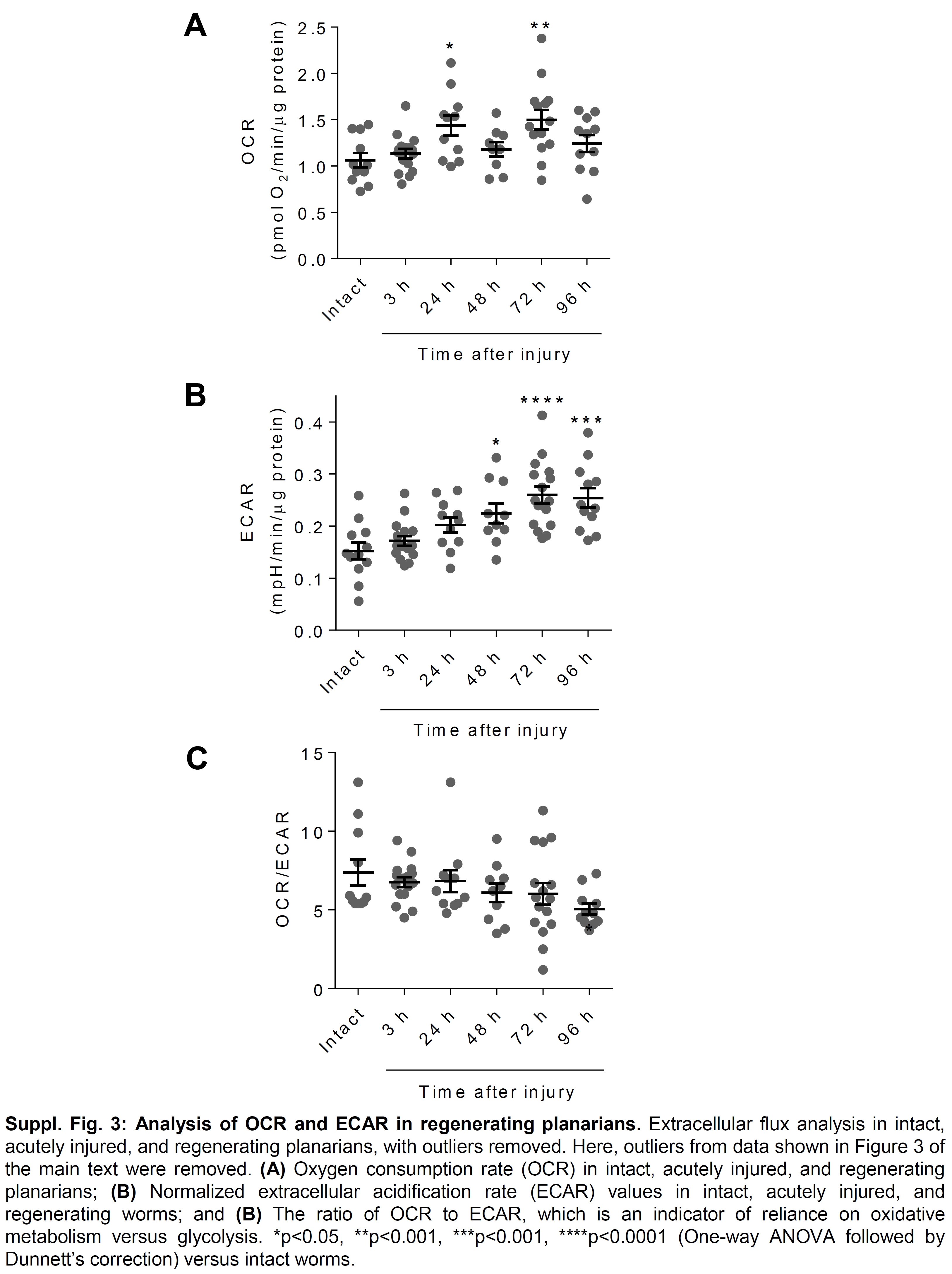

Supplement: Supplementary file 3 — Figure S3 [file REG2-5-78-s003.tif]

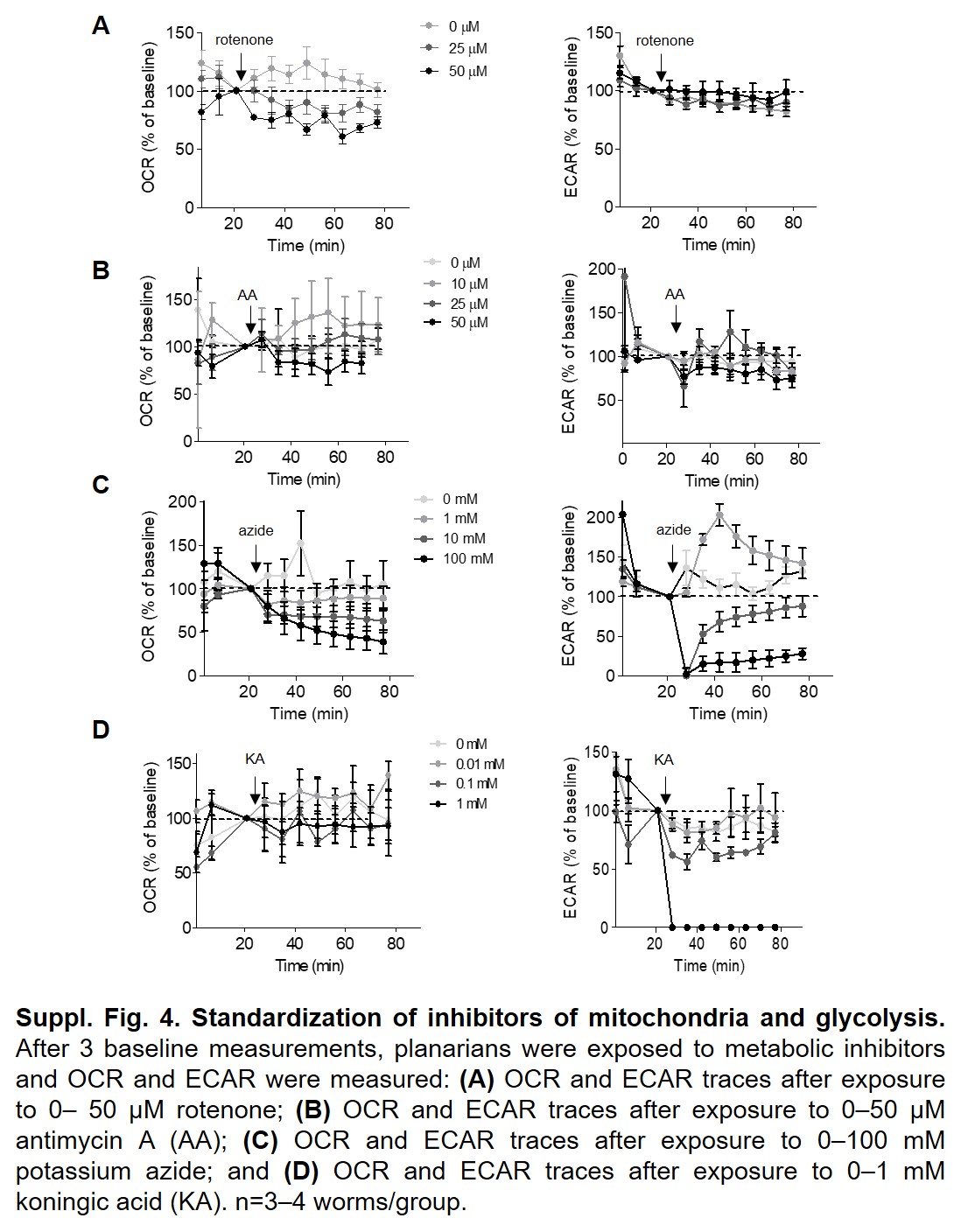

Supplement: Supplementary file 4 — Figure S4 [file REG2-5-78-s004.jpg]

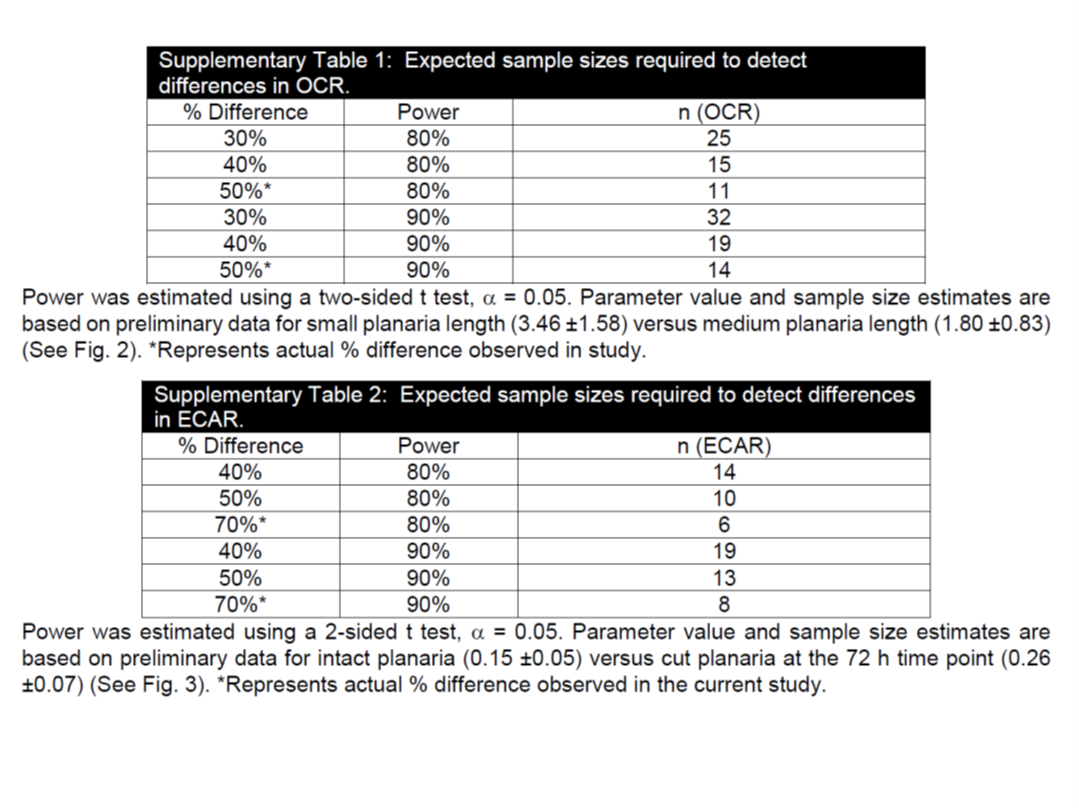

Supplement: Supplementary file 5 — Tables S1 and S2 [file REG2-5-78-s005.tif]
